# Activation of Wnt signaling by amniotic fluid stem cell-derived extracellular vesicles attenuates intestinal injury in experimental necrotizing enterocolitis

**DOI:** 10.1038/s41419-020-02964-2

**Published:** 2020-09-14

**Authors:** Bo Li, Carol Lee, Joshua S. O’Connell, Lina Antounians, Niloofar Ganji, Mashriq Alganabi, Marissa Cadete, Francesca Nascimben, Yuhki Koike, Alison Hock, Steven R. Botts, Richard Y. Wu, Hiromu Miyake, Adam Minich, Michael F. Maalouf, Elke Zani-Ruttenstock, Yong Chen, Kathene C. Johnson-Henry, Paolo De Coppi, Simon Eaton, Pekka Maattanen, Paul Delgado Olguin, Augusto Zani, Philip M. Sherman, Agostino Pierro

**Affiliations:** 1 Translational Medicine Program, The Hospital for Sick Children, Toronto, ON M5G 1X8 Canada; 2 Division of General and Thoracic Surgery, The Hospital for Sick Children, Toronto, ON M5G 1X8 Canada; 3 Developmental and Stem Cell Biology Program, The Hospital for Sick Children, Toronto, ON M5G 1X8 Canada; 4 Cell Biology Program, The Hospital for Sick Children, Toronto, ON M5G 1X8 Canada; 5 UCL Great Ormond Street Institute of Child Health, London, WC1N 1EH UK; 6 Biology Department, Burman University, Lacombe, AB T4L 2E5 Canada; 7 Department of Molecular Genetics, University of Toronto, Toronto, ON M5S 1A8 Canada; 8 Heart & Stroke Richard Lewar Centre of Excellence, Toronto, ON M5S 3H2 Canada; 9 Division of Gastroenterology, Hepatology and Nutrition, The Hospital for Sick Children, Toronto, ON M5G 1X8 Canada; 10 Department of Laboratory Medicine and Pathobiology, Faculty of Medicine, University of Toronto, Toronto, ON M5S 1A8 Canada; 11 Faculty of Dentistry, University of Toronto, Toronto, ON M5G 1G6 Canada; 12 Department of Surgery, University of Toronto, Toronto, ON M5S 1A8 Canada

**Keywords:** Intestinal stem cells, Intestinal diseases, Translational research

## Abstract

Necrotizing enterocolitis (NEC) is a devastating intestinal disease primarily affecting preterm neonates and causing high morbidity, high mortality, and huge costs for the family and society. The treatment and the outcome of the disease have not changed in recent decades. Emerging evidence has shown that stimulating the Wnt/β-catenin pathway and enhancing intestinal regeneration are beneficial in experimental NEC, and that they could potentially be used as a novel treatment. Amniotic fluid stem cells (AFSC) and AFSC-derived extracellular vesicles (EV) can be used to improve intestinal injury in experimental NEC. However, the mechanisms by which they affect the Wnt/β-catenin pathway and intestinal regeneration are unknown. In our current study, we demonstrated that AFSC and EV attenuate NEC intestinal injury by activating the Wnt signaling pathway. AFSC and EV stimulate intestinal recovery from NEC by increasing cellular proliferation, reducing inflammation and ultimately regenerating a normal intestinal epithelium. EV administration has a rescuing effect on intestinal injury when given during NEC induction; however, it failed to prevent injury when given prior to NEC induction. AFSC-derived EV administration is thus a potential emergent novel treatment strategy for NEC.

## Introduction

Necrotizing enterocolitis (NEC) is one of the most devastating diseases in newborn infants, primarily affecting preterm and low birth weight neonates^1,2^. There is currently no specific medical treatment for infants with NEC, and surgical resection of necrotic bowel segments often leads to short bowel syndrome and intestinal failure^3^. Despite extensive research into the prevention of NEC and significant improvements in neonatal care over the last three decades, NEC mortality remains as high as 30% of affected babies^4^. The infants that survive the disease can suffer lifelong complications, including short bowel syndrome and neurological sequelae. As a result, innovative treatment strategies are required to be able to reduce morbidity and mortality of the disease.

Intestinal stem cells (ISC) mediate intestinal regeneration after injury to prevent further intestinal damage^5^. ISC depletion correlates with severe gut damage during NEC development^6^, and dietary agents that promote ISC expansion ameliorate NEC^5^. We recently demonstrated that ISC impairment in NEC is caused by defective Wnt signaling^7^, a pathway that plays a central role in the regulation of stem cells and tissue homeostasis^8,9^, including gastrointestinal epithelium^10^. To avoid the broader effects of Wnt activation that can lead to potential undesirable consequences including tumorigenesis, controlled Wnt activation is highly desirable to provide a treatment strategy for NEC.

Amniotic fluid provides enteral nutrition as well as immunoregulatory, antimicrobial, and growth-promoting factors for immature enterocytes in utero, which prepares the gut for the dramatic shift from an in utero to a postnatal environment^11–13^. Administration of amniotic fluid to preterm born neonatal piglets increases body weight and prompts postnatal development^14,15^. In addition, amniotic fluid attenuates the severity of experimental NEC by reducing gut inflammation^16^ and inhibiting Toll-like receptor (TLR)-4 mediated signaling^17^. These beneficial effects may be due to the presence of amniotic fluid stem cells (AFSC). Administration of AFSC improves animal survival, clinical status, gut morphological structure, and intestinal function in experimental NEC^18,19^. AFSC have many similarities with embryonic stem cells, which may explain their capacity for pluripotency and rapid growth^20^. In neonatal rats with experimental NEC, we reported that AFSC administration decreased apoptosis and mucosal inflammation, and increased cell proliferation and migration through a paracrine mechanism^18^. However, the mechanism(s) by which AFSC elicited their protective effects during intestinal regeneration during NEC remains unclear.

It has previously been shown that the medium in which AFSC were grown ameliorated NEC and that extracellular vesicles (EV) can be isolated from this conditioned medium (CM). EV act as intercellular messengers to attenuate intestinal injury in NEC^21^. However, the mechanism by which these EV attenuated experimental NEC injury is unknown. We hypothesize that EV released by AFSC produce Wnt and, consequently, regulate ISC to reduce intestinal damage in experimental NEC.

## Materials and methods

### Amniotic fluid stem cells

Amniotic fluid was harvested from pregnant Sprague Dawley rats on day 14.5 of gestation via aspiration of amniotic sacs using a 25-gauge needle. C-kit (CD-117) positive selected AFSC were cultured and characterized, as previously described^22,23^. Briefly, AFSC were cultured in α-MEM medium (Thermo Fisher, Waltham, MA) supplemented with 20% Chang medium C (Irvine Scientific, Santa Ana, CA), 15% fetal bovine serum (FBS) (Thermo Fisher, Waltham, MA), and 1% penicillin/streptomycin (Pen-strep) (Sigma-Aldrich, St. Louis, MO), at 37 °C in a humidified chamber with 5% CO_2_.

Porcupine (Porcn) protein is required for Wnt protein maturation and release^24^. To create Wnt-deficient AFSC, a genetic knockout model could be ideally employed to completely eradicate Wnt signaling in AFSC. However, multiple attempts using CRISPR did not prove feasible, due to the vital role of Wnt in maintaining AFSC viability. We used two chemical inhibitors to block Wnt signaling. Porcn inhibitors Wnt-C59 (10 mM, Cayman Chemical, Ann Arbor, MI) and IWP2 (10 mM, Cayman Chemical, Ann Arbor, MI) were added to AFSC culture medium and incubated for 18 h. Wnt activity was assessed with the Cignal TCF/LEF Reporter Assay Kit (Qiagen, Hilden, Germany) with dual-luciferase assays (E1910, Promega, Madison, WI) following manufacturer’s instructions. Wnt-deficient AFSC were then administered to experimental animals.

### CM and EV

CM and EV were harvested according to our previously published method^23^. Briefly, 2 ml of serum-free CM was harvested from 4 × 10^6^ AFSC after 24 h culture. ExoQuick (EXOQ5A-1, System Biosciences, Palo Alto, CA) was used to isolate EV from AFSC CM. ExoQuick was added to the sample (5:1) and incubated overnight at 4 °C. The mixture was then centrifuged for 30 m at 1500 × *g* and the pellet resuspended in 100–500 µl of phosphate-buffered saline (PBS). EV characterization was confirmed, as outlined previously^23^.

### Animals and NEC model

All animal experiments were approved by the Animal Care Committee at The Hospital for Sick Children (no. 32238 and 44032), and all methods performed according to guidelines and regulations. Pups were randomly assigned to each of the experimental groups to eliminate potential within-litter effects. Experiments were designed to include multiple mice per group in each of the independent experiments. Samples size was based on previous experience and variability of results in these experimental studies. All of the experiments were independently repeated three times.

Experimental pups undergoing NEC induction (number for each study reported below) were separated from their mothers, while controls remained with mothers to breastfeed (*n* = 10). NEC was induced in neonatal C57BL/6 mixed sex mice from postnatal day 5–9 by gavage feeding of hyperosmolar formula, exposure to hypoxia and oral LPS injection (day 6 and 7, 4 mg/kg) for 4 days^7^. At postnatal day 9, pups were sacrificed, and the ileum was harvested for further studies. Mice with GFP-labeled Lgr5^+^ ISC (Lgr5-EGFP-IRES-creERT2) were obtained from Jackson Laboratory (Sacramento, CA) to study the fate of ISC in pups after the induction of NEC.

Three studies were conducted using the experimental mouse NEC model described above. Study 1, on postnatal days 6 and 7, during NEC induction, mice received an intraperitoneal injection of either PBS (*n* = 16), 2 × 10^6^ AFSC (*n* = 10), or 2 × 10^6^ Wnt-deficient AFSC (C59, *n* = 6; IWP2, *n* = 6), as described previously^18^. Study 2, AFSC-derived EV (AFSC-EV, same amount EV derived from 200 µl CM from 2 × 10^6^ AFSC, *n* = 8), was administered to the pups at same time as AFSC. We have previously demonstrated that the amount of EV in the CM, and not the quantity of the protein or medium, is the critical component to determine the magnitude of effect^23^. Study 3, on postnatal days 3 and 4 prior to NEC induction, groups of mice received an intraperitoneal injection of either PBS (*n* = 8) or AFSC-EV (same amount EV derived from 200 µl CM from 2 × 10^6^ AFSC, *n* = 8). Injections were given prior to NEC induction to determine the potential of preventing NEC development and progression.

### Intestinal morphology analysis

Ileal tissue was embedded in paraffin, sectioned (5 µm) and counterstained with hematoxylin. Stained sections were provided to three blinded investigators for assessment, following an established NEC histopathological scoring system^25,26^. Mice with NEC grade ≥ 2 were considered NEC-positive.

### Tissue immunostaining

Cells were fixed using 4% paraformaldehyde and subsequently permeabilized with 0.1% Triton X-100. After blocking with 1% BSA, cells were incubated overnight at 4 °C with a primary antibody (1:500 dilution) for cell proliferation Ki67 (ab15580, Abcam, Cambridge, MA). Cells were then incubated with secondary antibodies (1:1000 dilution, A-11034, Invitrogen, Carlsbad, CA, USA) and DAPI (Vector Laboratories, Burlington, ON, Canada) for nuclei at room temperature for 2 h. The image was captured using a Nikon TE-2000 digital microscope equipped with a Hamamatsu C4742-80-12AG camera. The number of antibody-labeled cells was quantified from five separate images by three blinded investigators.

Similarly, sections of terminal ileum were immunostained for Ki67 (ab15580, Abcam, Cambridge, MA) using the same protocol as above. For subsequent reactions, a streptavidin–biotin complex peroxidase kit (LASB + Kit, Dako, Denmark) was used and slides analyzed the same way as mentioned above.

### Two-photon laser scanning microscopy (TPLSM)

Taking advantage of TPLSM, we established a unique technique to visualize GFP-labeled ISC and assess ISC activity in live mouse pups under various conditions. The protocol was adapted from previous studies^27,28^. Briefly, a Mai Tai One Box Ti: Sapphire Laser (Newport Corporation-Spectra-Physics Lasers Division, Mountain View, CA), mode locked at 910 nm, was used as an excitation source. The Mai Tai laser produced a light pulse of ~100 fs of width (80 MHz repetition rate), which was directed onto the sample through the objective lens (×20, water immersion). Data were analyzed using ZEN software (Carl Zeiss, Jena, Germany). Intestinal tissue was scanned using TPLSM from the villus apex to the bottom of the crypts, followed by the submucosal and muscular layers.

### RT-qPCR

RNA from the distal ileum was isolated with TRIzol (Invitrogen). Total RNA (1 µg) was reverse transcribed using qScript cDNA supermix (Quanta Biosciences, Gaithersburg). SYBR green-based RT-qPCR was performed using a CF384 C1000 Thermal Cycler (Bio-Rad) and Evagreen Supermix (Bio-Rad) using previously described primers and conditions for *IL-6* and *Lgr5*^5^. Data were analyzed using CFX Manager 3.1 (Bio-Rad). Results are from three independent experiments, each performed in triplicate. Expression levels were calculated by the ∆∆Ct method and normalized to reference housekeeping genes *GAPDH* and *RPL0*.

### Intestinal organoids

Murine small intestine-derived organoids were cultured according to a previous publication^7^. Briefly, small intestine was harvested from neonatal C57BL/6 mice and cut into small segments. Intestinal crypts were isolated by digestion with Gentle Cell Dissociation Reagent (Stemcell Technologies, Cambridge, MA) for 15 m and pelleted by centrifugation. Crypts were then resuspended in Matrigel (Corning, New York) and transferred into 24-well plates. After polymerization, mouse IntestiCult organoid growth medium (Stemcell Technologies, Cambridge, MA) supplemented with penicillin-streptomycin (100 U/ml) was overlaid on the gel in each well. Organoids were maintained at 37 °C in 5% CO_2_ with culture medium replaced every 2 days. Organoids derived from healthy mice at p9 were exposed to either AFSC or Wnt-deficient AFSC (1 × 10^2^ cells in 25–40 organoids in Matrigel per well,) for 7 days. In addition, intestinal organoids derived from NEC mice at p9 were used to study the effects of either exogenous Wnt (200 ng/ml) or AFSC-EVs, which were supplied to the culture medium for 7 days. Control organoids were exposed to PBS for 7 days. Organoids were imaged daily, and surface area was calculated using Image J software. RNA was extracted with Trizol and gene expression analysis was then performed.

### IEC-18 cells

The rat intestinal epithelial cell line (IEC-18, ATCC, Manassas, VA) was cultured, as previously reported^29^, and maintained in DMEM medium (Gibco) supplemented with 10% FBS and 1% Pen-strep. Coculture studies were performed by seeding IEC-18 cells (1 × 10^3^) in 24-well plates and AFSC (1 × 10^2^) in the 6.5 mm sized Transwells (Corning, Mississauga, ON, Canada), and then placing the Transwells on top of the 24-well plate.

### Wound healing assay

Confluent monolayers of IEC-18 cells in 24-well plates were scraped with a 200 µl tip head and cocultured with stem cells in Transwells for 24 h. A Nikon TE-2000 digital microscope equipped with a Hamamatsu C4742-80-12AG camera recorded images of the wound area. Cell migration was assessed by measuring the gap distance between the leading edge of cells at the wound edge using Image J software. A blinded observer calculated the total denuded area covered by migrated cells per unit width of wound.

### Data analysis and statistics

All the experiments were conducted by investigators blind to the group allocation during the experiment and/or when assessing the outcome. No data were excluded from analysis. All the results passed the normal distribution test (Kolmogorov–Smirnov test) and are presented as means ± SD. Groups were compared using unpaired Student’s *t* test and one-way ANOVA with Bonferroni-corrected post-hoc testing, as appropriate. *p* < 0.05 was considered statistically significant.

## Results

### AFSC rescued intestinal injury, restored epithelial regeneration, and increased Lgr5+ISC in experimental NEC

**Fig. 1:**
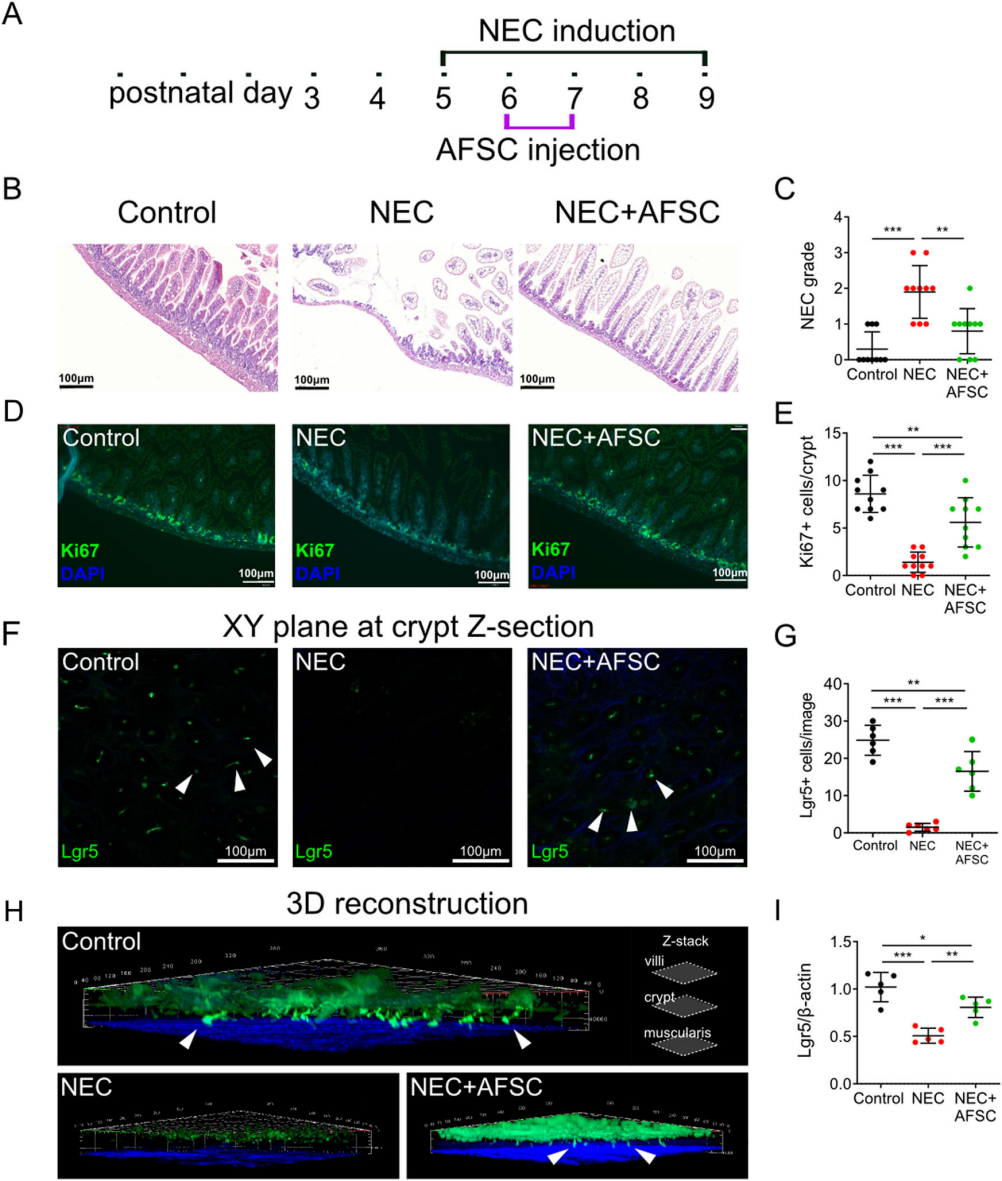
Amniotic fluid stem cells (AFSC) rescued intestinal injury, restored epithelial regeneration, and increased active intestinal stem cells (ISC). NEC induction was conducted on postnatal (p) days 5–9, with AFSC intraperitoneal injections given on p6 and p7 (**a**). Histopathology of ileal sections from mice administered with AFSC during necrotizing enterocolitis (NEC) induction showed normal villous structure when compared to NEC mice (**b**). Administration of AFSC during NEC significantly decreased histological scores (**c**). Intestinal epithelial proliferation (*Ki67*), which is reduced in NEC, was restored with AFSC administration (**d**, **e**). In vivo visualization of ISC after NEC induction showed a decrease in Lgr5^+^ ISC, some of which are denoted with white arrows, expression in NEC and restoration after AFSC treatment (**f**–**i**). *n* = 10 for each group (**b**–**e**) and *n* = 6 for each group (**f**–**i**). Data are presented as means ± SD. *p < 0.05; **p < 0.01; ***p < 0.001, using unpaired Student’s *t* test or one-way ANOVA with post-hoc tests as appropriate.

NEC induction was conducted from postnatal (p) day 5 to 9 with AFSC intraperitoneal injection given on p6 and p7, and animals sacrificed on p9 (Fig. 1a). Administration of AFSC attenuated NEC-induced gut injury in mice (Fig. 1b), as reflected by a decrease in NEC scoring grade (Fig. 1c). These findings were similar to previous findings in a rat model^18,19^. Intestinal epithelial proliferation marker, Ki67, was reduced in NEC (Fig. 1d) and expression was restored by the administration of AFSC (Fig. 1e). Using 3D-reconstructions of ileal tissue revealed a decrease in Lgr5+ ISC in NEC in vivo, with their restoration after AFSC exposure (Fig. 1f–i). Taken together, these results demonstrate that AFSC administration in NEC mice restored epithelial regeneration and increased the Lgr5+ ISC population.

### AFSC increased ISC and epithelial proliferation via Wnt signaling in intestinal organoids

**Fig. 2:**
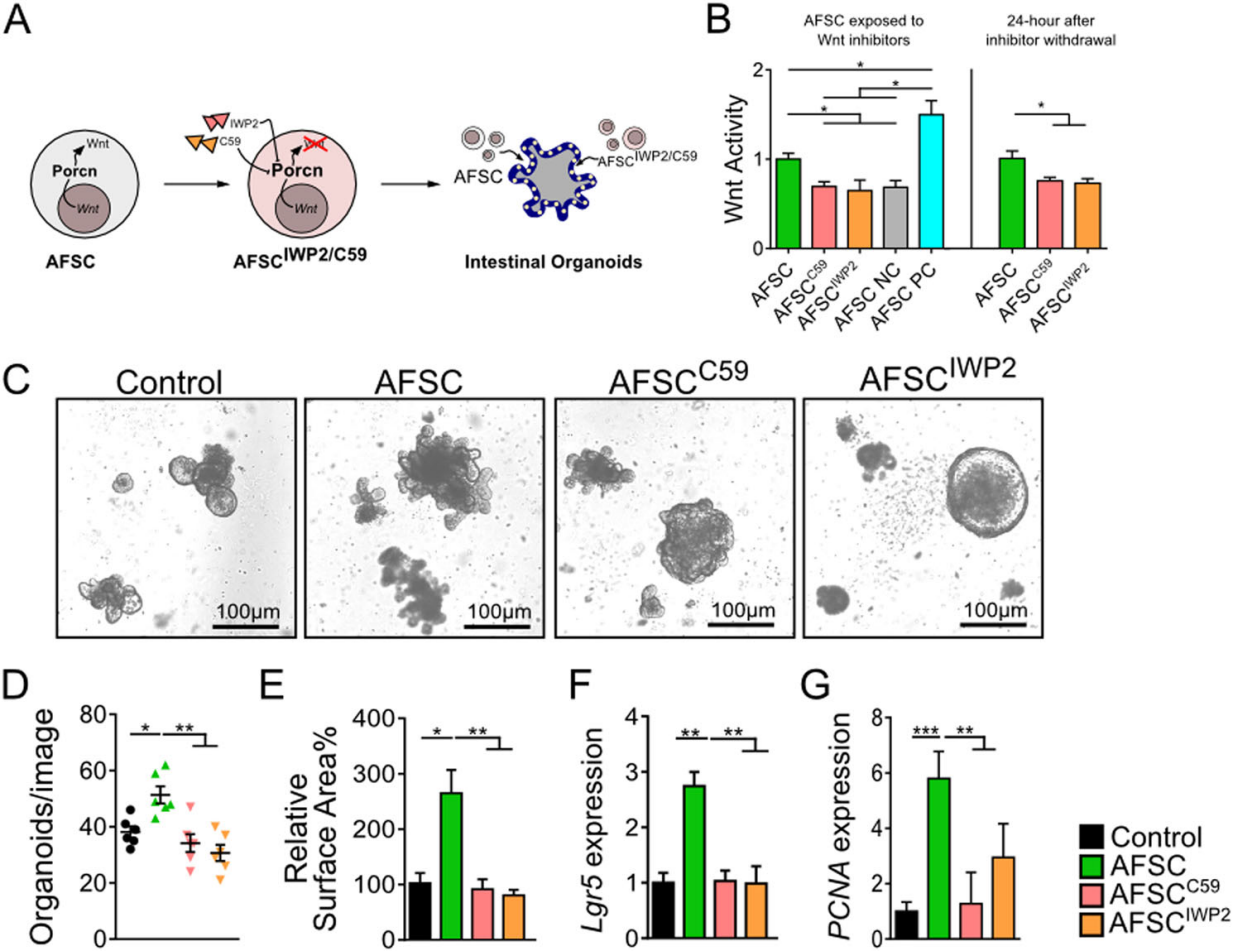
AFSC increased intestinal stem cells and epithelial proliferation via Wnt signaling in intestinal organoids. AFSC treated with two known inhibitors of Porcn, Wnt-C59, and IWP2 that are involved in Wnt maturation and release, were added to intestinal organoids (**a**). Wnt activity was reduced in Wnt-C59 and IWP2-treated AFSC with effects lasting ≥24 h after removal of the inhibitors, and a negative control (NC) and positive control (PC) were also included (**b**). After 7 days in culture, AFSC induced organoid growth and increased both the number and surface area of organoids, while Wnt-deficient AFSC failed to promote organoid growth (**c**–**e**). Organoids cocultured with AFSC had increased gene expression of the ISC marker *Lgr5* and proliferation marker *PCNA*, and this was not observed in Wnt-deficient AFSC (**f**, **g**). Data are presented as means ± SD. *n* = 6 for each group. *p < 0.05; **p < 0.01; ***p < 0.001, using unpaired Student’s *t* test or one-way ANOVA with post-hoc tests as appropriate.

As Porcn is important for maturation and release of Wnt protein^24^, two known inhibitors of Porcn, Wnt-C59 and IWP2, were used to inhibit AFSC (Fig. 2a). Wnt activity was significantly reduced in Wnt-C59 and IWP2-treated AFSC, which lasted at least 24 h after removal of the inhibitors (Fig. 2b). The decrease in Wnt activity caused by the presence of the inhibitors indicates that AFSC produce exogenous Wnt signaling mediators. To elucidate whether Wnt secreted by AFSC was required for restoration of intestinal regeneration, we cocultured mouse intestinal organoids with either AFSC (100 cells in 25–40 organoids per well) or Wnt-deficient AFSC (pretreated with C59 or IWP2). After 7 days in culture, the number of organoids and surface area was increased by AFSC, indicating that AFSC induced organoid growth, whereas Wnt-deficient AFSC failed to promote organoid growth (Fig. 2c–e). In addition, organoids cocultured with AFSC had increased gene expression of the ISC marker *Lgr5* and the proliferation marker *PCNA*, but this was not observed in the Wnt-deficient AFSC (Fig. 2f, g). Taken together, these findings indicate that the positive effects of AFSC on ISC and epithelial proliferation were mediated by Wnt signaling.

### Wnt signaling was necessary to attenuate intestinal damage during experimental NEC

**Fig. 3:**
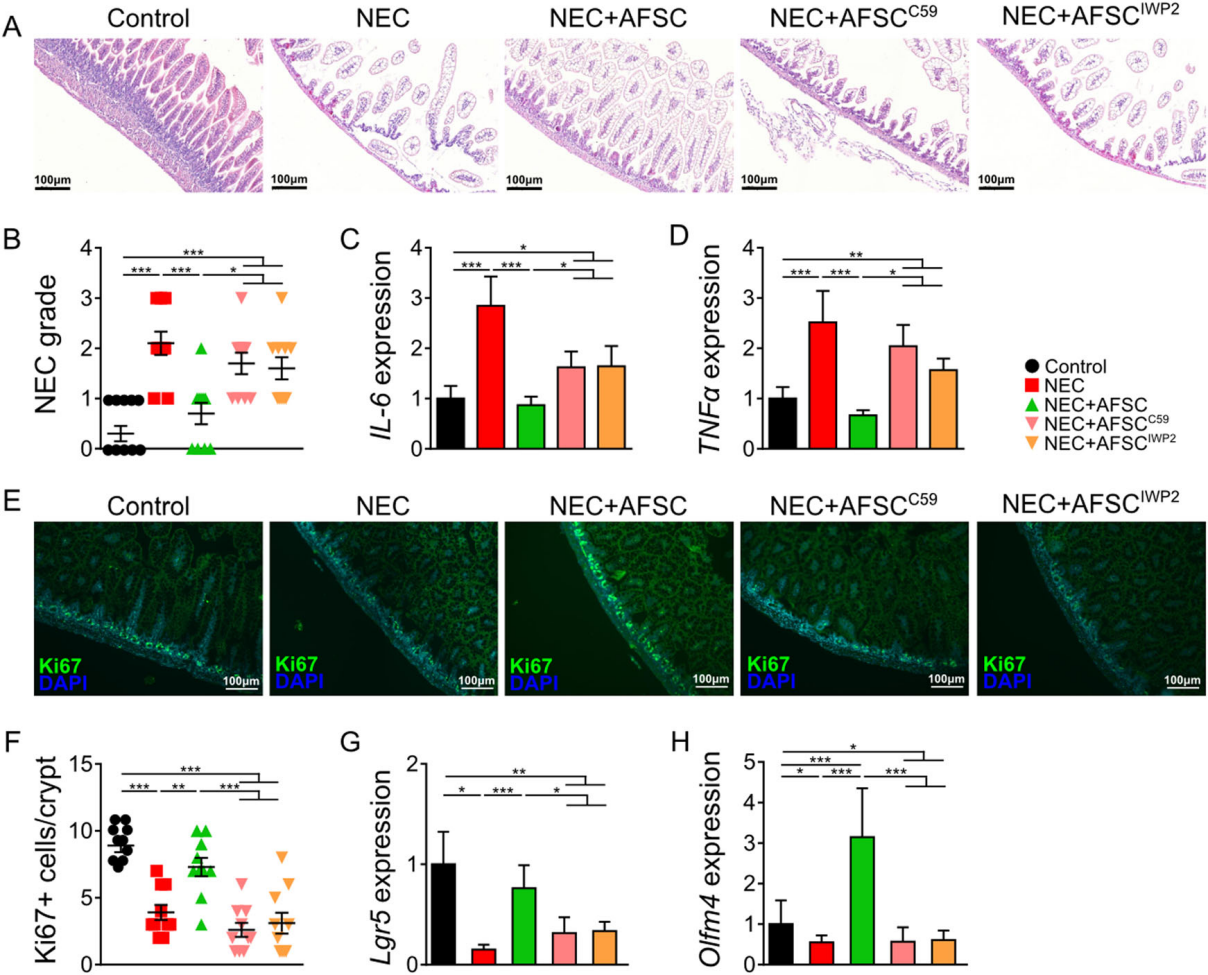
Wnt signaling was necessary to attenuate intestinal damage during experimental NEC. Ileal sections from NEC mice administered with C59 and IWP2-treated AFSC demonstrated increased villus damage as compared to NEC+AFSC (**a**, **b**). AFSC reduced NEC-induced intestinal expression of pro-inflammatory cytokines *IL-6* and *TNFα* (**c**, **d**), increased epithelial proliferation (*Ki67*) (**e**, **f**), and increased gene expression of ISC marker *Lgr5* and *Olfm4* (**g**, **h**). These changes were not evident with administration of Wnt-deficient AFSC (pretreated with C59 or IWP2). *n* = 10 for Control, NEC, NEC+AFSC, and *n* = 6 for NEC+AFSC^C59^, NEC+AFSC^IWP2^. Data are presented as means ± SD. *p < 0.05; **p < 0.01; ***p < 0.001, using unpaired Student’s *t* test or one-way ANOVA with post-hoc tests as appropriate.

To investigate whether Wnt secreted by AFSC was required for AFSC-mediated protection against NEC, we administered either Wnt-producing AFSC or Wnt-deficient AFSC (AFSC+C59, +IWP2) to mouse pups with NEC. Administration of Wnt-producing AFSC rescued intestinal epithelial morphology (Fig. 3a) and decreased the scoring of NEC grade (Fig. 3b). In addition, reduced intestinal expression of the pro-inflammatory cytokines *IL-6* and *TNFα* (Fig. 3c, d) was found in the AFSC-treated study group. After Wnt-producing AFSC administration, increased Ki67 epithelial proliferation (Fig. 3e, f) and increased gene expression of the ISC markers *Lgr5* and *Olfm4* (Fig. 3g, h). By contrast, these changes were not observed following administration of Wnt-deficient AFSC (AFSC pretreated with either C59 or IWP2) (Fig. 3a–h). Collectively, these findings demonstrate that Wnt signaling was required for the rescuing effects of AFSC on intestinal damage in experimental NEC.

### AFSC-secreted factors that increase ISC activity and Wnt pathway activation in both healthy and injured gut epithelia

**Fig. 4:**
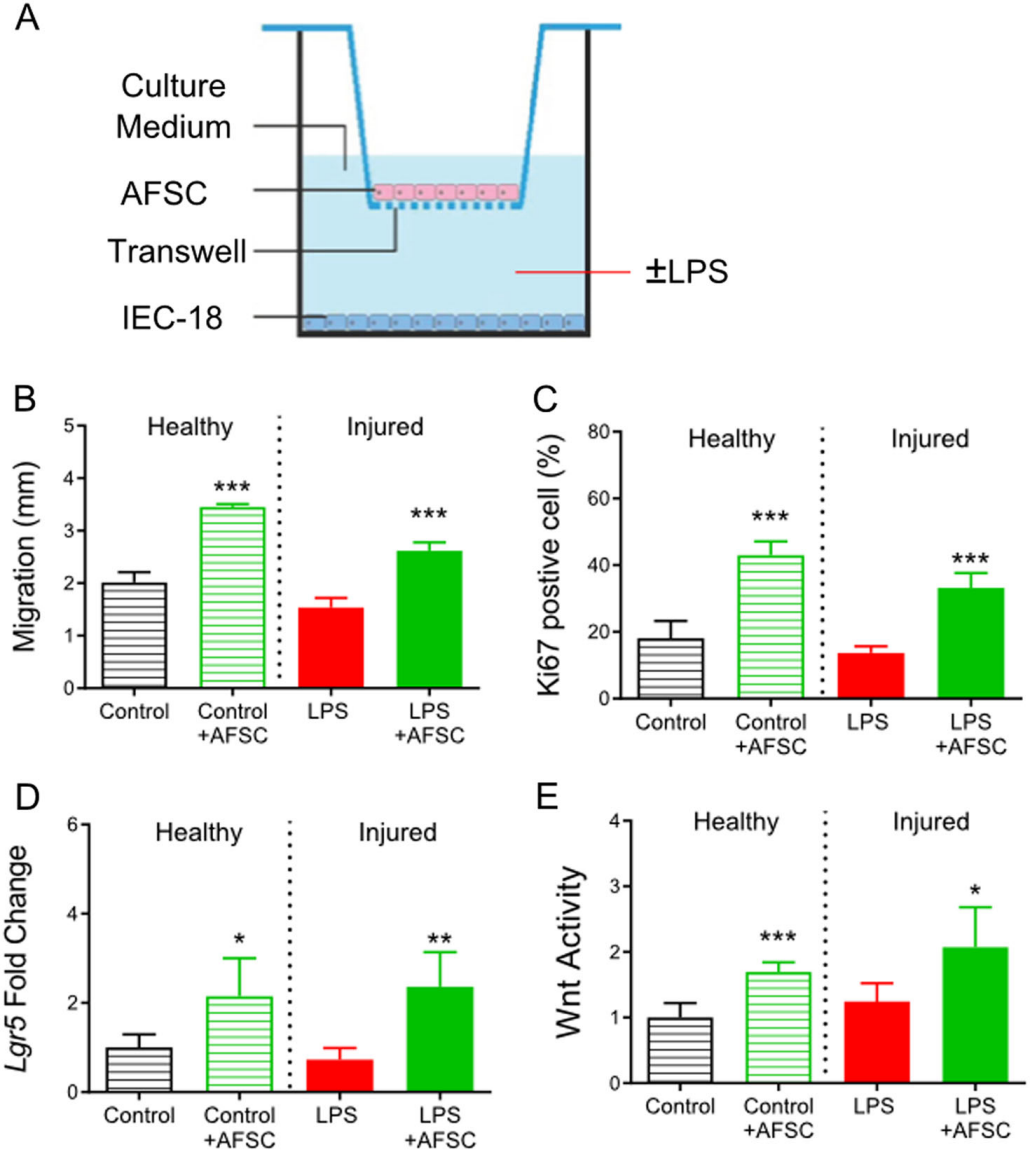
AFSC-secreted factors that increased intestinal stem cell activity and Wnt pathway activation in both healthy and injured intestinal epithelial cells. Transwells were used to coculture AFSC with intestinal epithelial cell line (IEC-18) cells (**a**). AFSC increased IEC migration to the apical compartment (**b**), intestinal cell proliferation (**c**), intestinal stem cell activity (**d**), and Wnt activity (**e**) during wound healing in both normal “healthy” and LPS-induced injury conditions. *n* = 6 for each group. Data are presented as means ± SD. *p < 0.05; **p < 0.01; ***p < 0.001, using unpaired Student’s *t* test or one-way ANOVA with post-hoc tests as appropriate.

To study the biological function of factors secreted by AFSC, we studied intestinal epithelial cells (IEC-18) under a normal “healthy” condition, with no LPS-induced injury, and IEC-18 exposed to LPS-induced injury. This set of experiments was performed using the Transwell system to coculture AFSC with IEC-18 cells, preventing direct contact between the cells, in the “healthy” and injured conditions (Fig. 4a). AFSC increased intestinal epithelial cell migration (Fig. 4b), proliferation (Fig. 4c), stem cell activity (Fig. 4d), and Wnt activity (Fig. 4e) as a response to wound healing in both healthy and injured conditions. These findings indicated that factors released from AFSC are as beneficial as whole AFSC and that direct contact of AFSC is not required to promote wound healing.

### AFSC-EV rescued organoids from NEC–injured intestinal tissue

**Fig. 5:**
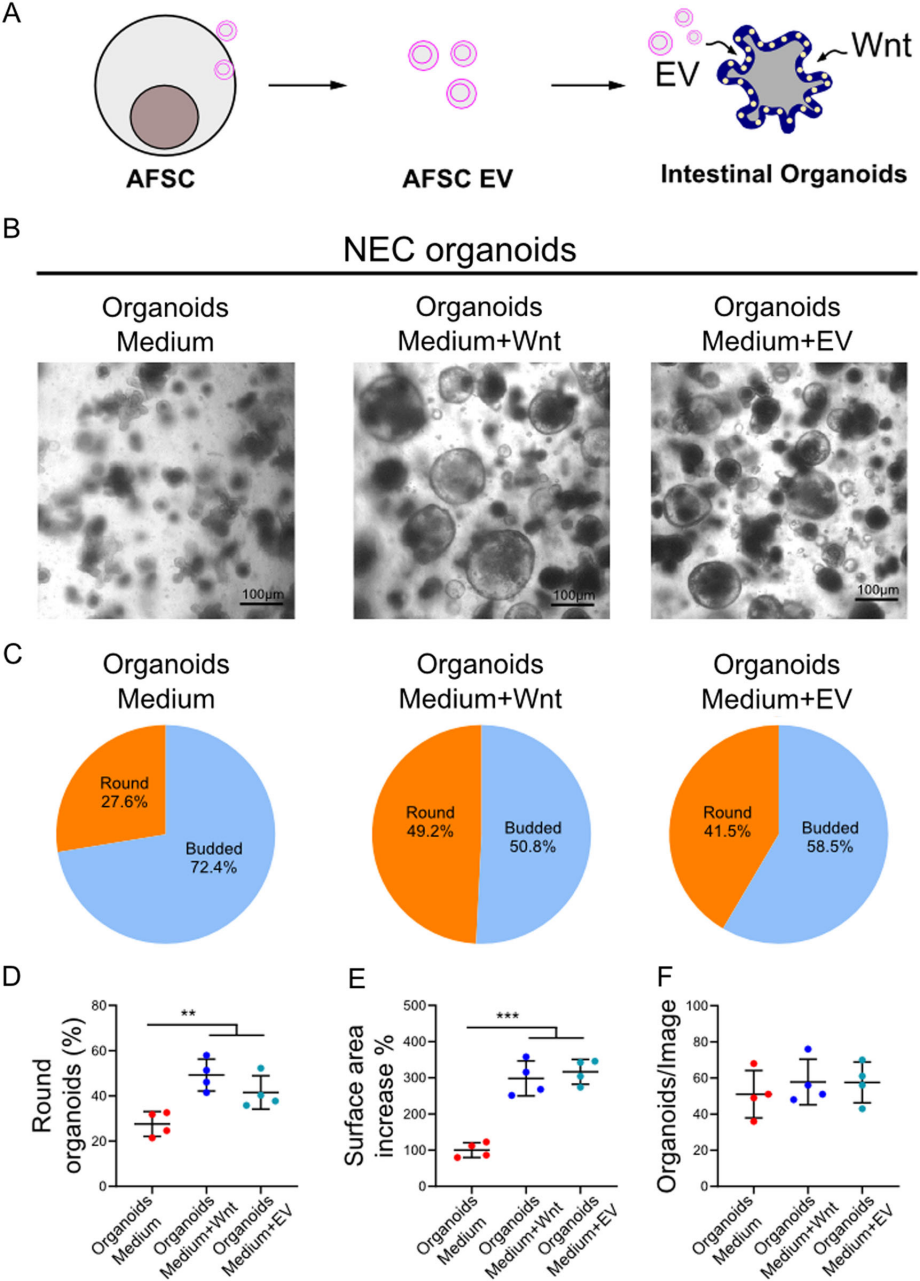
AFSC-derived EV rescued organoids derived from NEC–injured intestinal tissue in a similar manner to Wnt. EV derived from AFSC cultured media were harvested and used in NEC organoids (**a**). AFSC-derived EV added to culture medium increased organoid growth in a similar manner to exogenous Wnt enriched culture medium (**b**, **c**). NEC organoids treated with exogenous Wnt and EV also displayed more spherical shape, higher surface area, and increased numbers as compared to controls (**d**–**f**). *n* = 4 for each group. Data are presented as means ± SD. *p < 0.05; **p < 0.01; ***p < 0.001, using unpaired Student’s *t* test or one-way ANOVA with post-hoc tests as appropriate.

EV derived from AFSC-CM were isolated and characterized (Fig. 5a, Fig. S1). Compared to culture medium alone, intestinal organoids were larger following the addition of either Wnt or AFSC-EV (Fig. 5b). In addition, a larger percentage of the organoids were still round in the Wnt-rich and EV-rich media compared to control medium, which is indicative of maintained pluripotency. By contrast, the organoids grown in the normal culture medium were more budded, indicative of differentiation and loss of stemness (Fig. 5c–f). Taken together, these findings show that NEC organoids treated with AFSC-EV show a similar phenotype to the Wnt-rich medium.

### AFSC-EV improved intestinal growth in NEC-induced intestinal injury

**Fig. 6:**
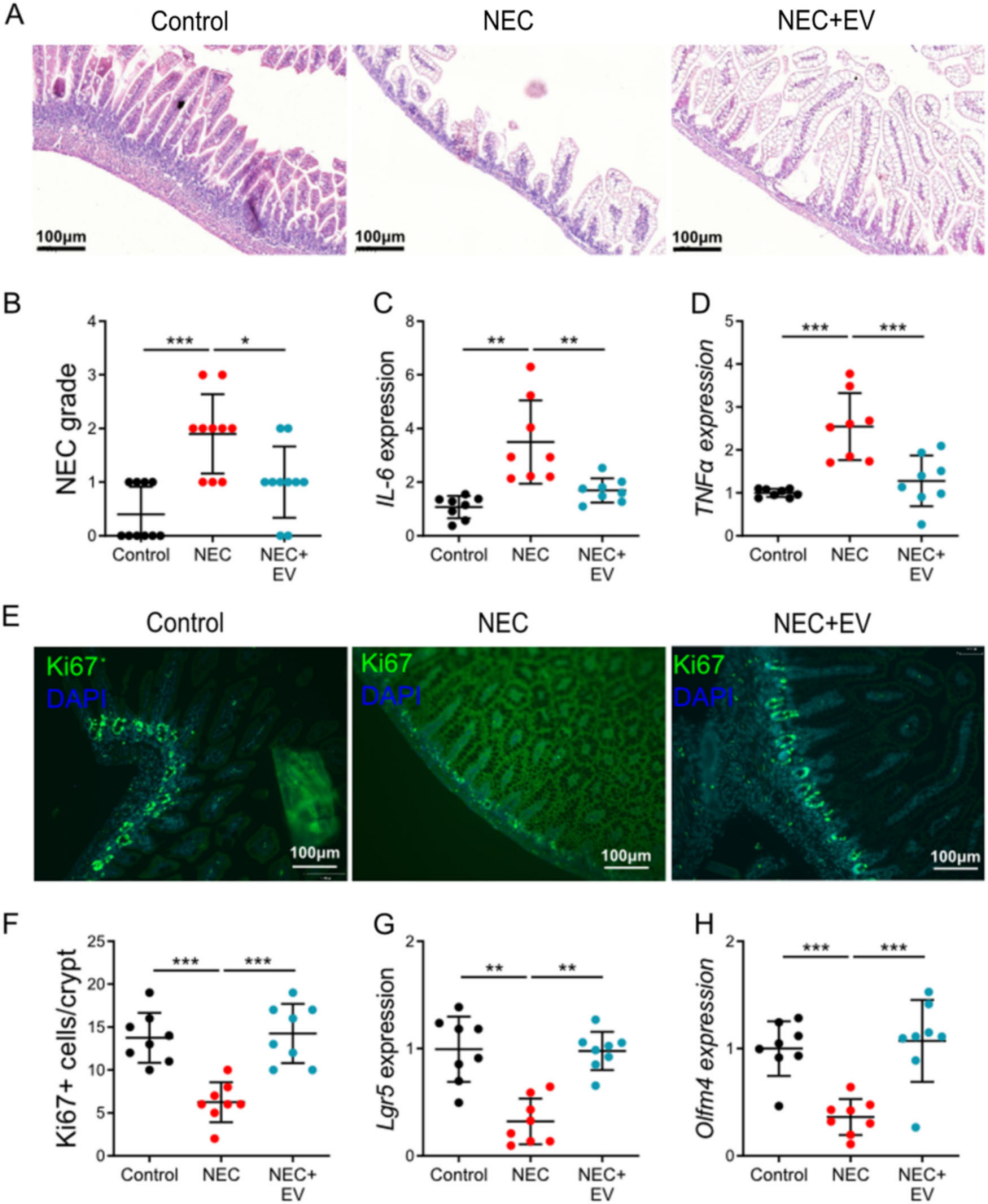
AFSC-derived EV improved intestinal growth in NEC-induced intestinal injury. Administration of EV derived from AFSC-conditioned medium on P6-P7 during NEC progression improved intestinal histology (**a, b**), and reduced IL-6 (**c**) and TNFα expression (**d**). There was also increased *Ki67* (**e**, **f**), *Lgr5* (**g**), and *Olfm4* (**h**) expression with EV administration relative to NEC alone. *n* = 8 for each group. Data are presented as means ± SD. *p < 0.05; **p < 0.01; ***p < 0.001, using unpaired Student’s *t* test or one-way ANOVA with post-hoc tests as appropriate.

Since EV derived from AFSC-CM were beneficial in NEC-derived intestinal organoids, we next investigated whether EV derived from AFSC-CM could alleviate intestinal injury in a murine model of NEC. EV derived from AFSC-CM were administered on P6-P7 during the induction of experimental NEC. NEC+EV mice had improved intestinal histology (Fig. 6a, b), reduced IL-6 and TNFα expression (Fig. 6c, d), and increased *Ki67*, *Lgr5, and Olfm4* expression (Fig. 6e–h) relative to the NEC group of mice not receiving EV from AFSC. The administration of AFSC-EV prior to the induction of NEC resulted in increased epithelium proliferation but did not reduce intestinal injury as indicated by the NEC severity score and intestinal inflammation as demonstrated by the IL-6 level (Fig. S2). These findings indicate that the timing of AFSC-EV administration is crucial as these EV can promote recovery from NEC intestinal damage but are not able to prevent its occurrence.

## Discussion

In this study, we have demonstrated that AFSC rescued NEC-induced intestinal injury by restoring epithelial regeneration and increasing Lgr5+-intestinal stem cells. In addition, we found that this was accomplished in a Wnt-dependent manner, and that both AFSC and EV derived from AFSC provided their beneficial effects on intestinal injury by stimulating the Wnt pathway. These findings have been demonstrated by (i) in vitro studies of IEC-18 cells or intestinal organoids derived from mouse pup intestine and (ii) in vivo experimental studies in a mouse pup model of NEC. Our results collectively indicate that EV derived from AFSC could be used as a potential new treatment in human infants with NEC, while avoiding some potential concerns associated with administering stem cells directly.

AFSC are associated with a reduced incidence and lessened severity of experimental NEC. The findings presented in this study, and in previous work^7,18^, demonstrate that AFSC differentially express genes of the Wnt/β-catenin pathway, which regulate intestinal epithelial stem cell function and cell migration. Beneficial effects from stem cell administration may be due to paracrine signaling, which can mediate intestinal injury in NEC. Indeed, we found that AFSC are effective in treating experimental NEC via modulation of stromal cells^18^. Although Wnt was not completely abolished in the current study setting, we demonstrated that Wnt was significantly attenuated for up to 48 h following the administration of Wnt inhibitors. We further showed that AFSC which were blunted in their ability to secrete Wnt displayed no beneficial effects on intestinal regeneration, thus demonstrating that AFSC protection from NEC occurs in a Wnt-dependent manner. This is in line with current literature reporting that the highly conserved Wnt pathway across species seems to be broadly implicated in processes that involve tissue homeostasis and regeneration^30–32^.

In our study, LPS was used to induce injury in intestinal epithelial cells (IEC-18). Consistent with previous studies, LPS inhibited cell migration and proliferation^33–35^, but did not impair Lgr5 expression and regeneration of intestinal epithelial cells^35^. In the current study, we demonstrate that administration of AFSC increases stem cell activity (Lgr5 expression), enhancing regeneration and repair in healthy and injured intestinal epithelium (Fig. 4). It has been previously demonstrated that LPS induces pro-inflammatory signaling and injury in enterocytes through the stimulation of TLR4^34–36^. Therefore, in our study, we did not explore further the LPS-mediated TLR4 activation.

Previous studies have not investigated the capacity of AFSC-secreted EV to protect against intestinal injury when administered either prior to the onset or during the progression of intestinal injury. In this study, we demonstrated that AFSC-secreted EV protected against intestinal injury and mucosal inflammation during NEC in a Wnt-dependent manner, which is similar to what was observed with AFSC. We have previously demonstrated that AFSC localize to sites of injury and inflammation in the developing intestine and are retained within these areas^18,37^. AFSC are potentially attracted via cell surface receptor activation to areas of intestinal damage indicating responsiveness to inflammatory and chemical mediators. This would be in keeping with what has been proposed for mesenchymal stem cell migration and their effects on tissue repair^38^. Even when administered prior to the onset of injury during the commencement of gut inflammation, AFSC can migrate and localize to the intestine and prevent the development of NEC. AFSC-secreted EV may not localize to sites of injury, thereby reducing their ability to impact areas of damaged intestine.

Based on our findings, both AFSC and AFSC-secreted EV act in a Wnt-dependent manner to similarly reduce inflammation and increase stem cell activity to rescue intestinal injury. AFSC, in general, are interesting cells to pursue novel medical investigations and treatment due to their lack of tumorigenicity when injected into immune-compromised animals, making them attractive cells for clinicians and researchers in the field of regenerative medicine and for severe diseases requiring cellular repopulation like NEC^39–41^. However, AFSC continue to raise some fear with respect to immunogenicity, and EV can help alleviate this concern^42^. Although EV presents a very interesting potential treatment option for NEC, deciphering how to ensure that administered EV are delivered to the site of injury can be challenging. More research into the delivery of EV to the area of injury and dosing are required.

Our findings highlight the important functional role that AFSC play in promoting cellular regeneration and mitigating NEC-induced damage in intestinal tissue, organoids, and live animals. AFSC can be readily and safely collected, cultured, and expanded^22,43,44^. Hence, there is great interest in their use for intestinal diseases such as NEC as well as their potential role in regenerative medicine.

In summary, our novel findings indicate that experimental NEC induces disruption of ISC and cell proliferation. These derangements can be rescued by the administration of exogenous Wnt produced from either intact AFSC or AFSC-EV. Accordingly, these EV could be used as a potential management consideration for prematurely born infants at risk of developing NEC as avoiding some of the potential risk associated with stem cell administration.

## Supplementary information

Figure S1

Figure S2

Supplementary Figure Legends

Video S1: TPLSM control sample

Video S2: TPLSM NEC sample

Video S3: TPLSM NEC+AFSC sample

Video S4: TPLSM 3-D construct of control sample

Video S5: TPLSM 3-D construct of NEC sample

Video S6: TPLSM 3-D construct of NEC+AFSC sample
