# Supplementary figures and images for: Activation of Wnt signaling by amniotic fluid stem cell-derived extracellular vesicles attenuates intestinal injury in experimental necrotizing enterocolitis
Source: Cell Death Dis. 2020 Sep 14;11(9):750. doi: 10.1038/s41419-020-02964-2 (PMC7490270; doi:10.1038/s41419-020-02964-2)

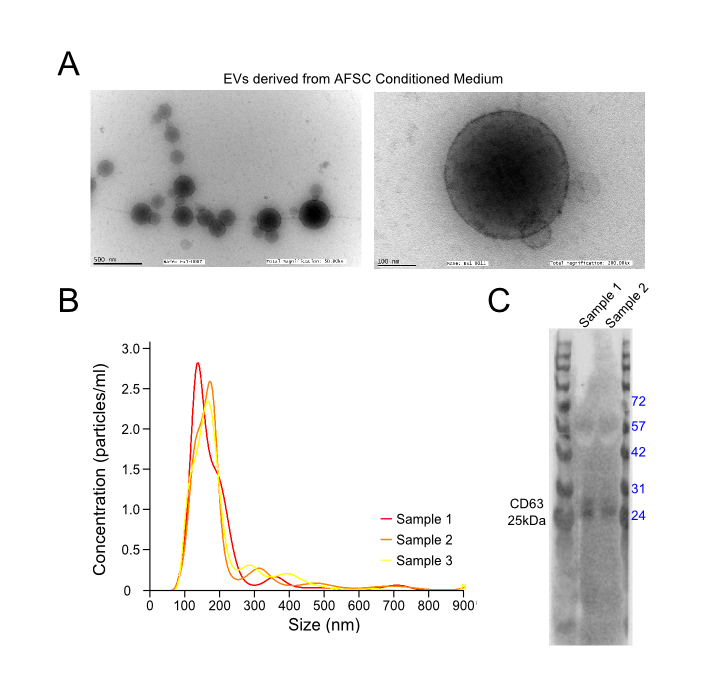

Supplement: Supplementary file 1 — Figure S1 [file 41419_2020_2964_MOESM1_ESM.tif]

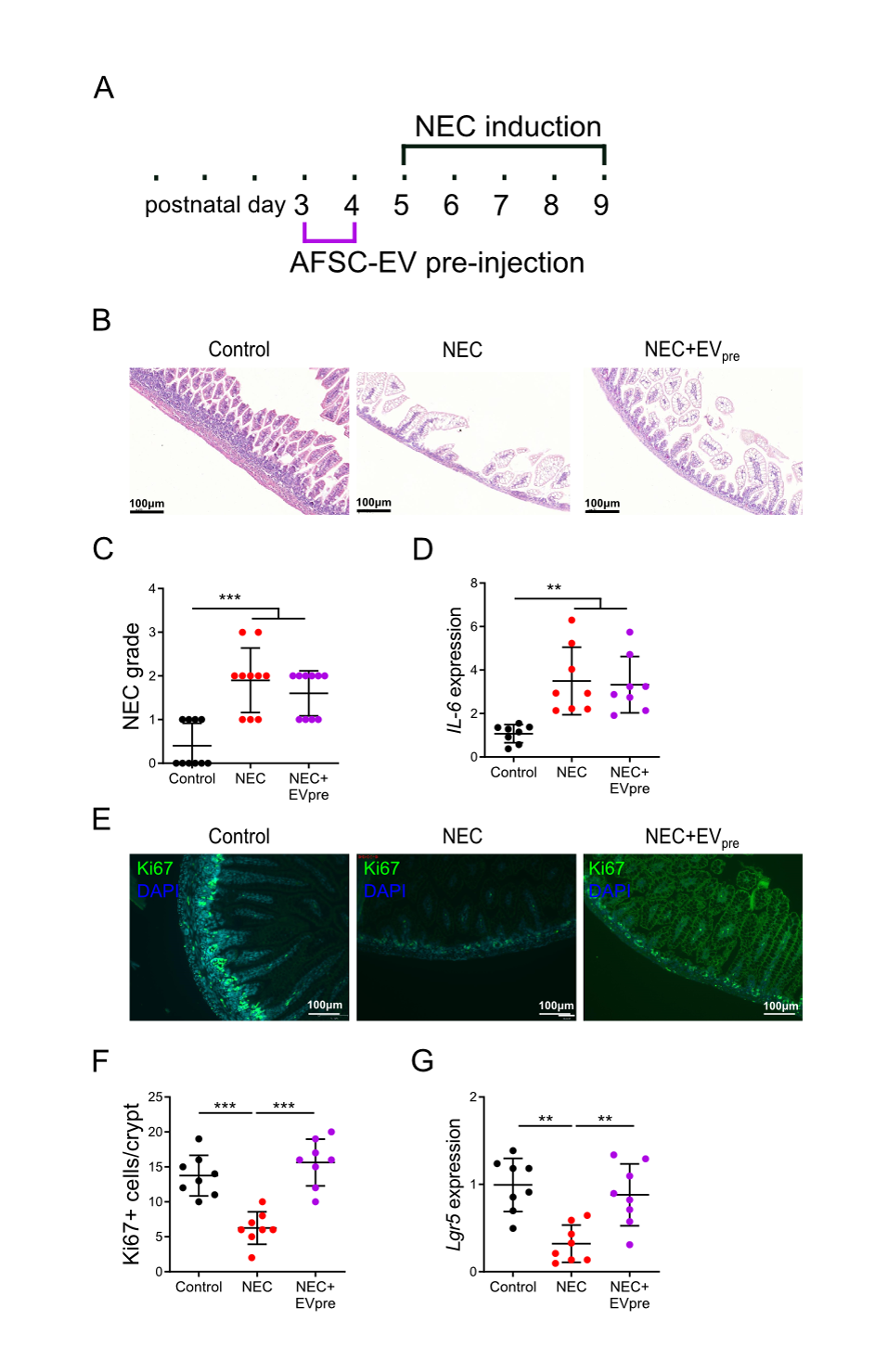

Supplement: Supplementary file 2 — Figure S2 [file 41419_2020_2964_MOESM2_ESM.tif]
