# Supplementary material for: Activation of Wnt signaling by amniotic fluid stem cell-derived extracellular vesicles attenuates intestinal injury in experimental necrotizing enterocolitis
Source: Cell Death Dis. 2020 Sep 14;11(9):750. doi: 10.1038/s41419-020-02964-2 (PMC7490270; doi:10.1038/s41419-020-02964-2)
Supplement: Supplementary file 3 — Supplementary Figure Legends [file 41419_2020_2964_MOESM3_ESM.docx]

**SUPPLEMENTRY FIGURE LEGENDS**

**Figure S1: Characterization of EV derived from AFSC conditioned medium.** Concentration and size distribution confirmed EV isolation and purification **(A, B)**. Size distribution was confirmed by western blot analysis **(C)**.

**Figure S2: EV administered before NEC onset did not prevent NEC progression or reduce NEC.**

EV derived from AFSC conditioned medium were administered on P3-P4 before NEC induction **(A)**. No notable improvement in the EV treated mice compared to the NEC mice with respect to histology **(B, C)**, IL-6 **(D)**, *Ki67* **(E, F)** or *Lgr5* **(G)** was observed. n=8 for each group. Data are presented as means ± SD. *p < 0.05; **p < 0.01; ***p < 0.001, using unpaired student’s t-test or one-way ANOVA with post-hoc tests as appropriate.

**SUPPLEMENTARY VIDEOS**

**Video S1: TPLSM control sample**

**Video S2: TPLSM NEC sample**

**Video S3: TPLSM NEC+AFSC sample**

**Video S4: TPLSM 3-D construct of control sample**

**Video S5: TPLSM 3-D construct of NEC sample**

**Video S6: TPLSM 3-D construct of NEC+AFSC sample**
